# Supplementary material for: Karyopherins remodel the dynamic organization of the nuclear pore complex transport barrier
Source: Nat Cell Biol. 2025 Dec 2;27(12):2089–101. doi: 10.1038/s41556-025-01812-9 (PMC12717009; doi:10.1038/s41556-025-01812-9)
Supplement: Supplementary file 1 — Supplementary Figs. 1,2 and Supplementary Tables 1–4. [file 41556_2025_1812_MOESM1_ESM.pdf]

nature cell biology

Supplementary information https://doi.org/10.1038/s41556-025-01812-9

Karyopherins remodel the dynamic organization of the nuclear pore complex transport barrier

In the format provided by the authors and unedited

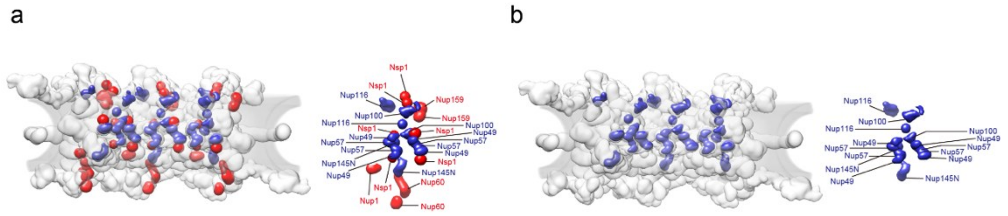

Supplementary Fig. 1 FG domain anchor sites on the NPC scaffold. a-b, Side view of three spokes showing the FG domain anchor sites for FxFG (red) and GLFG (blue) Nups in WT **(a)** and ΔFG NPCs **(b)**. Each view is accompanied by a single spoke (right) with specific FG domains labelled.

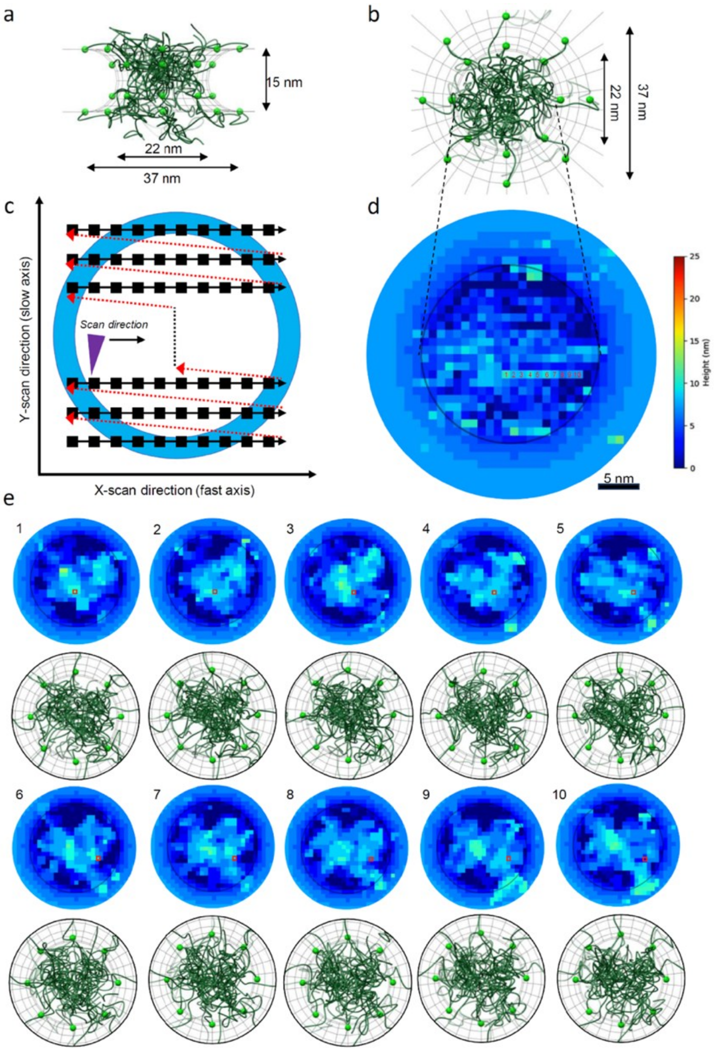

Supplementary Fig. 2 Assessing HS-AFM data asynchronicity. a-b, Snapshots of a Brownian dynamics (BD) simulation presenting a side view (**a**) and top view (**b**) of Nsp1FG tethered to a 22 nm-diameter toroidal nanopore. Green and light green represent FG domains and their anchoring sites, respectively. **c,** A schematic of the rasterization process of the HS-AFM. The HS-AFM tip moves along the fast-scan axis while collecting data points indicated by black square boxes. As the tip reaches the rightmost pixel, it goes back to the leftmost pixel while moving up by one pixel in the slow-scan axis. **d-e,** A simulated HS-AFM image (**d**) of the BD model obtained by a simulated raster scan of the HS-AFM tip. The outermost blue ring corresponds to the toroidal wall. The time required to capture one pixel of data along the X-axis in the HS-AFM image is 10 ns per pixel, which is equivalent to the duration of one BD "snapshot". Pixel values indicated by numbers in the image, are derived from the ground truth HS-AFM images in (**e**). **e,** The first and third rows depict non-rasterized HS-AFM images (ground truth) computed from the BD snapshots in the second and forth rows, respectively.

Supplementary Table 1. Properties of *S. cerevisiae* FG Nups

S. cerevisiae	AAs in disordered domain[a]	Predicted hydrodynamic diameter [1a]	Cohesiveness[a]	Copy number[b]	Number of FG repeats[c]
Nup159	387-1071	13.4	Non-cohesive	16	25
Nup100	1-610	9.8	Cohesive	16	44
Nup100	611-800	7.4	Non-cohesive		
Nup116	172-764	9.8	Cohesive	16	42
Nup116	765-960	7.6	Non-cohesive		
Nup42	1-382	8.2	Cohesive	8	18
Nup49	1-251	5.0	Cohesive	32	16
Nsp1	1-186	6.2	Cohesive	48	33
Nsp1	187-617	12.4	Non-cohesive		
Nup57	1-255	6.8	Cohesive	32	16
Nup145N	1-242	5.0	Cohesive	16	11
Nup145N	243-433	6.4	Non-cohesive		
Nup1	220-797	13.6	Non-cohesive	8	17
Nup1	798-1076	7.2	Cohesive		
Nup2	160-800	12.6	Non-cohesive	16	12
Nup60	389-539	6.6	Non-cohesive	16	4

[a]Based on Yamada et al., 2010.

[b]Based on Kim et al., 2018.

[c]Number of FG repeats is taken from Uniprot (https://www.uniprot.org/)

AAs = amino acids

Supplementary Table 2. FG repeat and FG domain concentrations in yeast WT, ΔFG and Nsp1FGx2 NPCs and NuPODs

	Volume	FG repeat		FG domain	
	nm^3	mg/ml	mM	mg/ml	mM
**Isolated WT yNPC[a]**	~157,963[b]	~13	~53	~96	~2.3
**Isolated ΔFG yNPC[a]**	~157,963[b]	~6	~27	~40	~1.1
**Isolated Nsp1FGx2 yNPC[a]**	~157,963[b]	~16	~69	~124	~2.8
**In situ WT yNPC[a]**	~194,190[b]	~10	~43	~78	~1.9
**NuPOD**	~84780	~5	~21	~39	~0.6

Note: The volume corresponds to the CT and is calculated within the region that is capped by the top and the bottom of the scaffold.

[a]Numbers of FG repeats are taken from Uniprot (https://www.uniprot.org/). FG domain copy numbers are based on Kim et al., 2018 and Huang et al., 2020.

[b]Isolated yNPC and in situ yNPC volumes are calculated from Kim et al., 2018 and Akey et al., 2022, respectively.

Supplementary Table 3. Strains used in this study.

Name	SOURCE	IDENTIFIER
MATα ade2-1 ura3-1 his3-11,15 trp1-1 leu2-3,112 can1-100	Euroscarf	W303α (parental)
MATα ade2-1 ura3-1 his3-11,15 trp1-1 leu2-3,112 can1-100 MLP1-PPX-ProteinA::HIS5	[2]	Mlp1ppxPrA
trp1-1 lys2 ura3 leu2 his3 Flag-LoxP-nsp1DFGDFxFG T7-LoxP-Nup1DFxFG myc-LoxP-nup2DFxFG myc-LoxP-nup60DFxF HA-LoxP-Nup42DFG my-LoxP-Nup159DFG	[3]	SWY3062
trp1-1 lys2 ura3 leu2 his3 Flag-LoxP-nsp1DFGDFxFG T7-LoxP-Nup1DFxFG myc-LoxP-nup2DFxFG myc-LoxP-nup60DFxF HA-LoxP-Nup42DFG my-LoxP-Nup159DFG MLP1-PPX-ProteinA::HIS5	This study	DeltaFG, Mlp1-PPX-PrA
MATα ade2-1 ura3-1 his3-11,15 trp1-1 leu2-3,112 can1-100 nsp1::NatMX4 pRS416-NSP1	This study	DeltaNsp1
MATα ade2-1 ura3-1 his3-11,15 trp1-1 leu2-3,112 can1-100 nsp1::NatMX4 p410-ADH1pr-NSP1(1-591)-NSP1(2-565)-NSP1(592-824)::KanMX MLP1-PPX-ProteinA::HIS5	This study	Nsp1FGx2, Mlp1-PPX-PrA
MATα ade2-1 ura3-1 his3-11,15 trp1-1 leu2-3,112 can1-100 KAP95-link-ymNeongreen::URA3	This study	W303, Kap95-mNeongreen
MATα ade2-1 ura3-1 his3-11,15 trp1-1 leu2-3,112 can1-100 MLP1-PPX-ProteinA::HIS5 KAP95-link-ymNeongreen::URA3	This study	Mlp1ppxPrA, Kap95-mNeongreen
MATα ade2-1 ura3-1 his3-11,15 trp1-1 leu2-3,112 can1-100 nsp1::NatMX4 p410-ADH1pr-NSP1(1-591)-NSP1(2-565)-NSP1(592-824)::KanMX MLP1-PPX-ProteinA::HIS5 KAP95-link-ymNeongreen::URA3	This study	Nsp1FGx2, Mlp1-PPX-PrA, Kap95-mNeongreen
MATa his3Δ1 leu2Δ0 met15Δ0 ura3Δ0 pGAL1-GFP-Nup100FG	This study	GFP-Nup100FG
MATa his3Δ1 leu2Δ0 met15Δ0 ura3Δ0 pGAL1-GFP-Nup153FG	This study	GFP-Nup153FG
MATa his3Δ1 leu2Δ0 met15Δ0 ura3Δ0 pGAL1-GFP-Nup116FG	This study	GFP-Nup116FG
MATa his3Δ1 leu2Δ0 met15Δ0 ura3Δ0 pGAL1-GFP-Nup159SFG	This study	GFP-Nup159SFG
MATa his3Δ1 leu2Δ0 met15Δ0 ura3Δ0 pGAL1-GFP-Nsp1FG	This study	GFP-Nsp1FG
MATa his3Δ1 leu2Δ0 met15Δ0 ura3Δ0 pGAL1-GFP-Nup60FG	This study	GFP-Nup60FG
MATa his3Δ1 leu2Δ0 met15Δ0 ura3Δ0 nup100-GFP::His3MX6	ThermoFisher	Nup100-GFP
MATa his3Δ1 leu2Δ0 met15Δ0 ura3Δ0 nup133-GFP::His3MX6	ThermoFisher	Nup133-GFP
MATa his3Δ1 leu2Δ0 met15Δ0 ura3Δ0 nup159-GFP::His3MX6	ThermoFisher	Nup159-GFP
MATa his3Δ1 leu2Δ0 met15Δ0 ura3Δ0 nsp1-GFP::His3MX6	ThermoFisher	Nsp1-GFP
MATa his3Δ1 leu2Δ0 met15Δ0 ura3Δ0 nup60-GFP::His3MX6	ThermoFisher	Nup60-GFP
MATa his3Δ1 leu2Δ0 met15Δ0 ura3Δ0 nup1-GFP::His3MX6	ThermoFisher	Nup1-GFP
MATα ade2-1 ura3-1 his3-11,15 trp1-1 leu2-3,112 can1-100 TEFF-mCherry-WALP-HDEL::Leu2 pGAL1-GFP-Nup100FG	This study	mCherry-WALP-HDEL, GFP-Nup100FG
MATα ade2-1 ura3-1 his3-11,15 trp1-1 leu2-3,112 can1-100 MLP1-PPX-ProteinA::HIS5 TEFF-mCherry-WALP-HDEL::Leu2 pGAL1-GFP-Nup100FG	This study	Mlp1-PPX-PrA, mCherry-WALP-HDEL, GFP-Nup100FG
MATα ade2-1 ura3-1 his3-11,15 trp1-1 leu2-3,112 can1-100 nsp1::natMX4 p410-ADH1pr-NSP1(1-591)-NSP1(2-565)-NSP1(592-824)::KanMX MLP1-PPX-ProteinA::HIS5 TEFF-mCherry-WALP-HDEL::Leu2 pGAL1-GFP-Nup100FG	This study	Nsp1FGx2, Mlp1-PPX-PrA, mCherry-WALP-HDEL, GFP-Nup100FG
trp1-1 lys2 ura3 leu2 his3 Flag-LoxP-nsp1DeltaFGDeltaFxFG T7-LoxP-Nup1DeltaFxFG myc-LoxP-nup2DeltaFxFG myc-LoxP-nup60DeltaFxF HA-LoxP-Nup42DeltaFG my-LoxP-Nup159DeltaFG MLP1-PPX-ProteinA::HIS5 TEFF-mCherry-WALP-HDEL::Leu2 pGAL1-GFP-Nup100FG	This study	DeltaFG, Mlp1-PPX-PrA, mCherry-WALP-HDEL, GFP-Nup100FG

MATα ade2-1 ura3-1 his3-11,15 trp1-1 leu2-3,112 can1-100 pGAL1-mCherry-L-TM pGAL1-MG4	This study	W303, mCherry-L-TM, MG4
MATα ade2-1 ura3-1 his3-11,15 trp1-1 leu2-3,112 can1-100 nsp1::natMX4 p410-ADH1pr-NSP1(1-591)-NSP1(2-565)-NSP1(592-824)::KanMX pGAL1-mCherry-L-TM pGAL1-MG4	This study	Nsp1FGx2, mCherry-L-TM, MG4
trp1-1 lys2 ura3 leu2 his3 Flag-LoxP-nsp1DeltaFGDeltaFxFG T7-LoxP-Nup1DeltaFxFG myc-LoxP-nup2DeltaFxFG myc-LoxP-nup60DeltaFxF HA-LoxP-Nup42DeltaFG my-LoxP-Nup159DeltaFG pGAL1-mCherry-L-TM pGAL1-MG4	This study	DeltaFG, mCherry-L-TM, MG4

Supplementary Table 4. Plasmids used in this study.

Name	SOURCE	IDENTIFIER
pAG25	Euroscarf	dominant marker gene deletion cassette: pAgTEF1-natMX4-tAgTEF1
PLPC19	[4]	pRS416-NSP1
pYX242-SV40NLS-GFP-PrA	[5]	#202 (http://ncdir.org)
p410 Adh1 Nsp1FGx2	This study	p410 plasmid with NSP1(1-591)-(2-565)-(592-824) under control of yeast Adh1 promoter and terminator
pGAL1-GFP-Nup100FG	This study	Adapted from pACM021[6], GAL1 promoter, N-term GFP tag Nup100FG domain, His3+ selection marker
pGAL1-GFP-Nup116FG	This study	As above, Nup116FG domain
pGAL1-GFP-Nup153FG	This study	As above, Nup153FG domain
pGAL1-GFP-Nup159SFG	This study	As above, Nup159SFG domain
pGAL1-GFP-Nsp1FG	This study	As above, Nsp1FG domain
pGAL1-GFP-Nup60FG	This study	As above, Nup60FG domain
pGAL1-GFP-Nup100FG	This study	Adapted from pACM021, GAL1 promoter, N-term GFP tag Nup100FG domain, Ura3+ selection marker
pTEFF-mCherry-WALP-HDEL::Leu	This study	mCherry N-term tagged NE/ER marker protein under constitutive TEFF promoter, Leu2 selection marker
pGAL1-mCherry-L-TM	[6]	pUG36, mCherry-linker(180)-TM under GAL1 promoter URA selection marker
pGAL1-MG4	[7]	MBP-4xGFP under GAL1 promoter His selection marker
pFA6a-link-ymNeongreen-URA3	Addgene[8]	Addgene #168059

References

1. Molines, A.T. *et al.* Physical properties of the cytoplasm modulate the rates of microtubule polymerization and depolymerization. *Dev. Cell* **57**, 466-479.e466 (2022).
2. Kim, S.J. *et al.* Integrative structure and functional anatomy of a nuclear pore complex. *Nature* **555**, 475-482 (2018).
3. Strawn, L.A., Shen, T.X., Shulga, N., Goldfarb, D.S. & Wente, S.R. Minimal nuclear pore complexes define FG repeat domains essential for transport. *Nature Cell Biology* **6**, 197-206 (2004).
4. Colombi, P., Webster, B.M., Frohlich, F. & Lusk, C.P. The transmission of nuclear pore complexes to daughter cells requires a cytoplasmic pool of Nsp1. *J Cell Biol* **203**, 215-232 (2013).
5. Leslie, D.M., Timney, B., Rout, M.P. & Aitchison, J.D. Studying nuclear protein import in yeast. *Methods* **39**, 291-308 (2006).
6. Meinema, A.C. *et al.* Long unfolded linkers facilitate membrane protein import through the nuclear pore complex. *Science* **333**, 90-93 (2011).
7. Popken, P., Ghavami, A., Onck, P.R., Poolman, B. & Veenhoff, L.M. Size-dependent leak of soluble and membrane proteins through the yeast nuclear pore complex. *Molecular Biology of the Cell* **26**, 1386-1394 (2015).
8. Botman, D., de Groot, D.H., Schmidt, P., Goedhart, J. & Teusink, B. In vivo characterisation of fluorescent proteins in budding yeast. *Sci Rep* **9**, 2234 (2019).
