# Supplementary material for: Karyopherins remodel the dynamic organization of the nuclear pore complex transport barrier
Source: Nat Cell Biol. 2025 Dec 2;27(12):2089–101. doi: 10.1038/s41556-025-01812-9 (PMC12717009; doi:10.1038/s41556-025-01812-9)
Supplement: Supplementary file 2 — Reporting Summary [file 41556_2025_1812_MOESM2_ESM.pdf]

nature portfolio

Corresponding author(s): Roderick Lim & Michael Rout

Last updated by author(s): Oct 3, 2025

Reporting Summary

Nature Portfolio wishes to improve the reproducibility of the work that we publish. This form provides structure for consistency and transparency in reporting. For further information on Nature Portfolio policies, see our Editorial Policies and the Editorial Policy Checklist.

Statistics

For all statistical analyses, confirm that the following items are present in the figure legend, table legend, main text, or Methods section.

n/a	Confirmed	
☐	☒	The exact sample size (*n*) for each experimental group/condition, given as a discrete number and unit of measurement
☐	☒	A statement on whether measurements were taken from distinct samples or whether the same sample was measured repeatedly
☐	☒	The statistical test(s) used AND whether they are one- or two-sided<br>*Only common tests should be described solely by name; describe more complex techniques in the Methods section.*
☒	☐	A description of all covariates tested
☒	☐	A description of any assumptions or corrections, such as tests of normality and adjustment for multiple comparisons
☐	☒	A full description of the statistical parameters including central tendency (e.g. means) or other basic estimates (e.g. regression coefficient) AND variation (e.g. standard deviation) or associated estimates of uncertainty (e.g. confidence intervals)
☐	☒	For null hypothesis testing, the test statistic (e.g. *F*, *t*, *r*) with confidence intervals, effect sizes, degrees of freedom and *P* value noted<br>*Give P values as exact values whenever suitable.*
☒	☐	For Bayesian analysis, information on the choice of priors and Markov chain Monte Carlo settings
☒	☐	For hierarchical and complex designs, identification of the appropriate level for tests and full reporting of outcomes
☒	☐	Estimates of effect sizes (e.g. Cohen's *d*, Pearson's *r*), indicating how they were calculated

Our web collection on statistics for biologists contains articles on many of the points above.

Software and code

Policy information about availability of computer code

Data collection	Igor Pro (6.3), Chemidoc imaging system (BioRad), softWoRx (Cytiva, Version 7.0.0), PicoScope (7.1.50)
Data analysis	Python (2.7, 3.9), ImageJ (1.53c, 1.53t, 1.54p), Relion (3.1.2), easyFRAP software (web version), cryoSPARC (4), SpectroMine (2.8.210609.47784), Igor Pro (8.0), Prism software (GraphPad, 9.1.0, 10.2.3, 10.4.2, 10.6.1)

For manuscripts utilizing custom algorithms or software that are central to the research but not yet described in published literature, software must be made available to editors and reviewers. We strongly encourage code deposition in a community repository (e.g. GitHub). See the Nature Portfolio guidelines for submitting code & software for further information.

Data

Policy information about availability of data

All manuscripts must include a data availability statement. This statement should provide the following information, where applicable:

- Accession codes, unique identifiers, or web links for publicly available datasets
- A description of any restrictions on data availability
- For clinical datasets or third party data, please ensure that the statement adheres to our policy

Yeast strains and plasmids generated in this study will be distributed without restriction upon request.
Source data are provided with this study:
Source Data 1 - All numerical values used to generate plots.

Source Data 2 - Unprocessed images of gels and blots.

HS-AFM data files are available in Zenodo under the identifier: 10.5281/zenodo.15684361.
Mass spectrometry data have been deposited in PRIDE with the primary accession code PXD069104.
Source data have been provided in Source Data.
Software scripts and simulation data are available at https://github.com/ravehlab/HS-AFM-Simulation
Codes used for CP tracking are available at https://github.com/toshiya-kozai/Single-Particle-tracking-on-AFM-kymograph
All other data supporting the findings of this study are available on reasonable request by the lead contacts, Michael Rout (rout@rockefeller.edu) and Roderick Lim (roderick.lim@unibas.ch).

Research involving human participants, their data, or biological material

Policy information about studies with human participants or human data. See also policy information about sex, gender (identity/presentation), and sexual orientation and race, ethnicity and racism.

Reporting on sex and gender	NA
Reporting on race, ethnicity, or other socially relevant groupings	NA
Population characteristics	NA
Recruitment	NA
Ethics oversight	NA

Note that full information on the approval of the study protocol must also be provided in the manuscript.

Field-specific reporting

Please select the one below that is the best fit for your research. If you are not sure, read the appropriate sections before making your selection.

☒ Life sciences ☐ Behavioural & social sciences ☐ Ecological, evolutionary & environmental sciences

For a reference copy of the document with all sections, see nature.com/documents/nr-reporting-summary-flat.pdf

Life sciences study design

All studies must disclose on these points even when the disclosure is negative.

Sample size	No statistical methods were used to determine sample size.
Data exclusions	No data exclusions were made.
Replication	HS-AFM and TEM data were analyzed for structurally intact isolated WT or mutant NPCs. All replicates for in vivo assays were successful.
Randomization	Samples were not randomized in the experiments.
Blinding	Experiments were not blinded.

Reporting for specific materials, systems and methods

We require information from authors about some types of materials, experimental systems and methods used in many studies. Here, indicate whether each material, system or method listed is relevant to your study. If you are not sure if a list item applies to your research, read the appropriate section before selecting a response.

Materials & experimental systems

n/a	Involved in the study
☐	☒ Antibodies
☒	☐ Eukaryotic cell lines
☒	☐ Palaeontology and archaeology
☒	☐ Animals and other organisms
☒	☐ Clinical data
☒	☐ Dual use research of concern
☒	☐ Plants

Methods

n/a	Involved in the study
☒	☐ ChIP-seq
☒	☐ Flow cytometry
☒	☐ MRI-based neuroimaging

Antibodies

Antibodies used

Monoclonal antibody mouse Anti-NSP1 (1:5000 dilution) (Abcam, ab4641), Rabbit IgG affinity purified (Innovative Research, IRBIGGAP500MG), Monoclonal antibody mouse anti-GFP (1:2500 dilution) (Santa Cruz, sc-9996), Monoclonal antibody anti-mouse m-IgGκ BP-HRP (1:2500 dilution) (Santa Cruz, sc-516102), Monoclonal antibody mouse anti-mCherry (1:100 dilution) (ThermoFisher, (4B3)MA5-32977), Monoclonal antibody mouse anti-GFP (1:20 dilution) (Roche, 11814460001), Alexa Fluor 568-conjugated antibody anti-mouse (1:200 dilution) (ThermoFisher, A11004), Polyclonal antibody rabbit anti-eGFP (1:20 dilution) (ThermoFisher, CAB4211)

Validation

Antibodies were validated by the manufacturers. For Anti-NSP1, see https://www.abcam.com/products/primary-antibodies/nsp1-antibody-32d6-ab4641.html; for anti-GFP (Santa Cruz), see https://www.scbt.com/p/gfp-antibody-b-2; for anti-mouse m-IgGκ BP-HRP, see https://www.scbt.com/p/m-igg-kappa-bp-hrp; for anti-mCherry, see https://www.thermofisher.com/antibody/product/mCherry-Antibody-clone-4B3-Monoclonal/MA5-32977; for anti-GFP (Roche), see https://www.sigmaaldrich.com/CH/en/product/roche/11814460001?srsltid=AfmBOoo5-4C9eieJScKh75j3GNu1zIjf1U740_fR_PHBVxlkl7r4x09_; for Alexa568 anti-mouse, see https://www.thermofisher.com/antibody/product/Goat-anti-Mouse-IgG-H-L-Cross-Adsorbed-Secondary-Antibody-Polyclonal/A-11004; for anti-eGFP, see https://www.thermofisher.com/antibody/product/eGFP-Antibody-Polyclonal/CAB4211
