# Supplementary material for: Karyopherins remodel the dynamic organization of the nuclear pore complex transport barrier
Source: Nat Cell Biol. 2025 Dec 2;27(12):2089–101. doi: 10.1038/s41556-025-01812-9 (PMC12717009; doi:10.1038/s41556-025-01812-9)
Supplement: Supplementary file 13 — Unprocessed blots for Extended Data Figs. 1p, 5a and 6b. [file 41556_2025_1812_MOESM13_ESM.pdf]

ED Fig. 1p

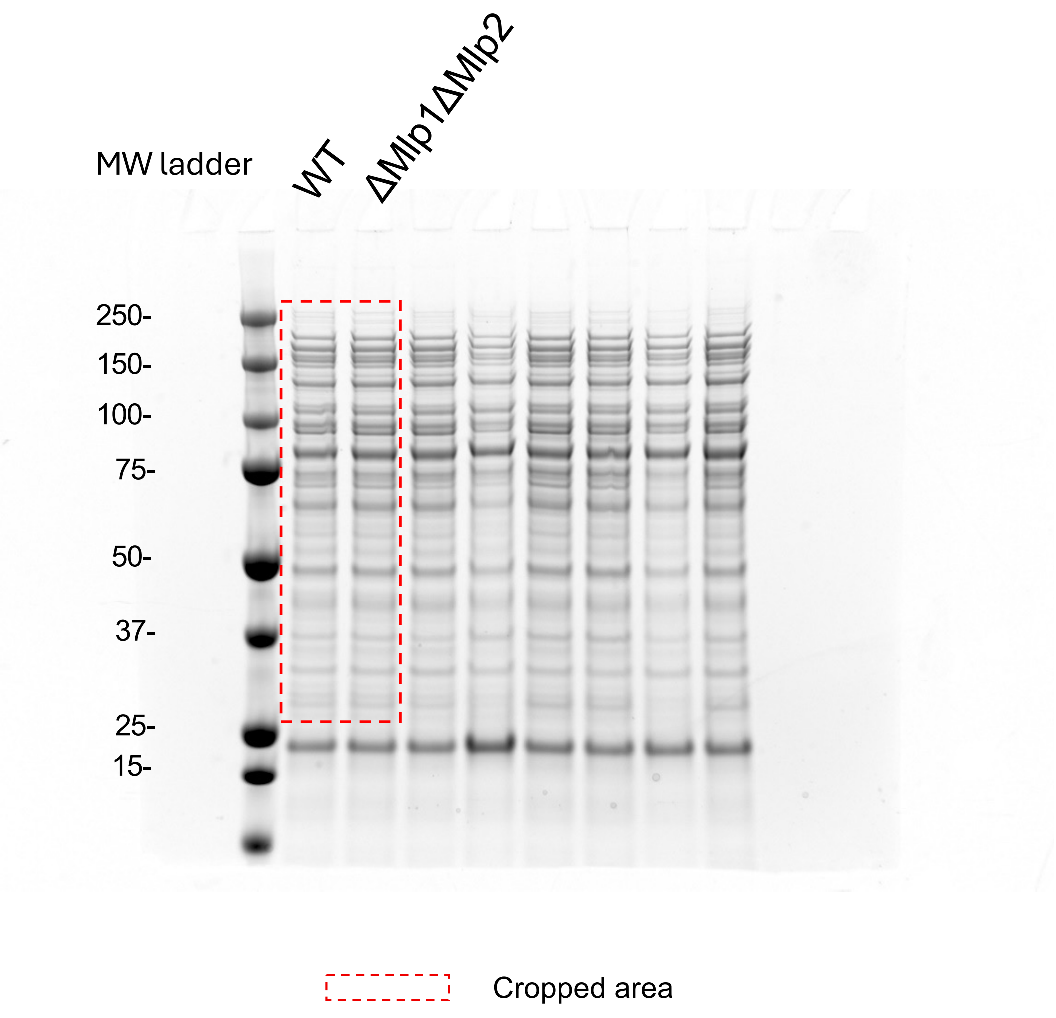
MW ladder
WT
ΔMlp1ΔMlp2
250-
150-
100-
75-
50-
37-
25-
15-
Cropped area

ED Fig. 5a (left panel)

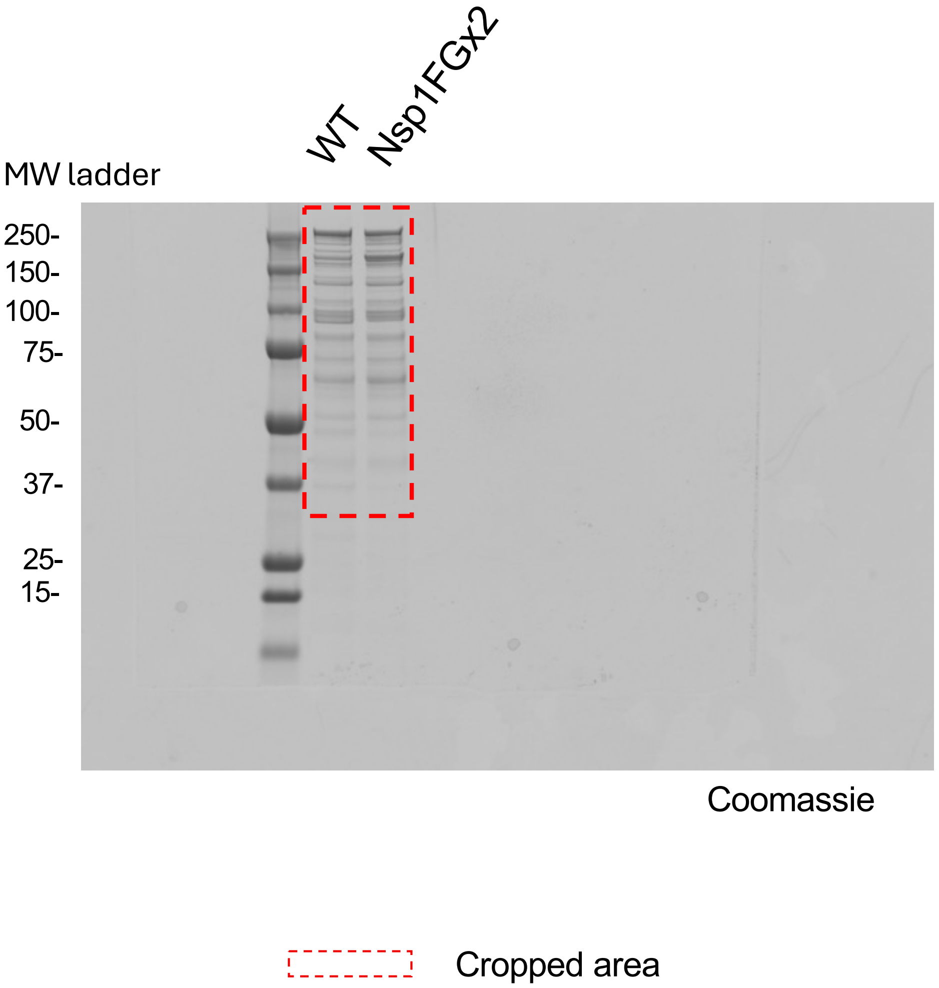

ED Fig. 5a (right panel)

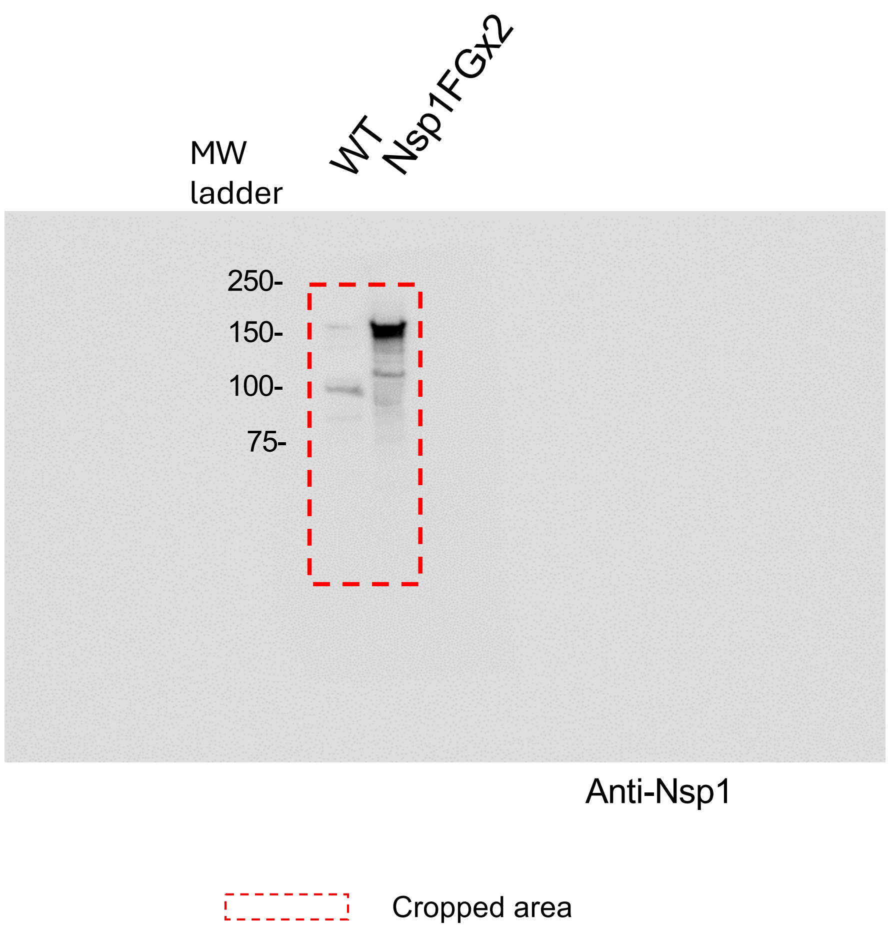

ED Fig. 6b

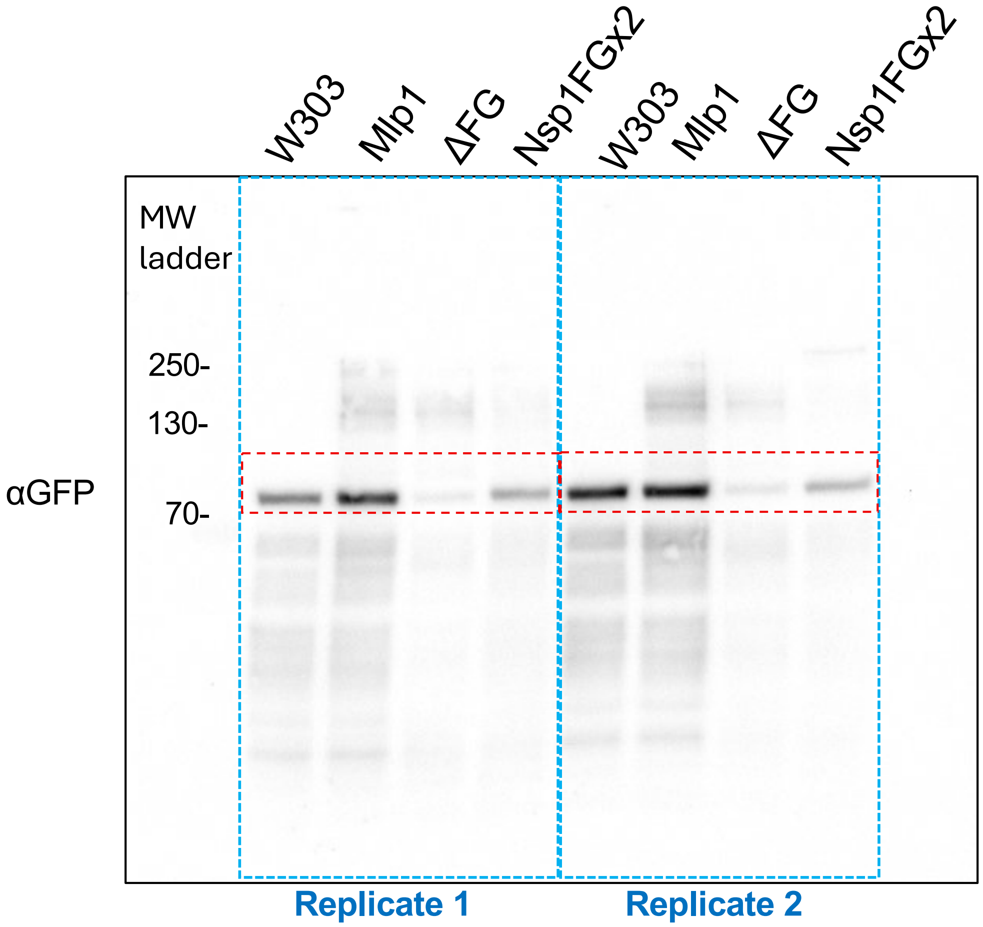

Area delimiting each replicate

Cropped area

ED Fig. 6b

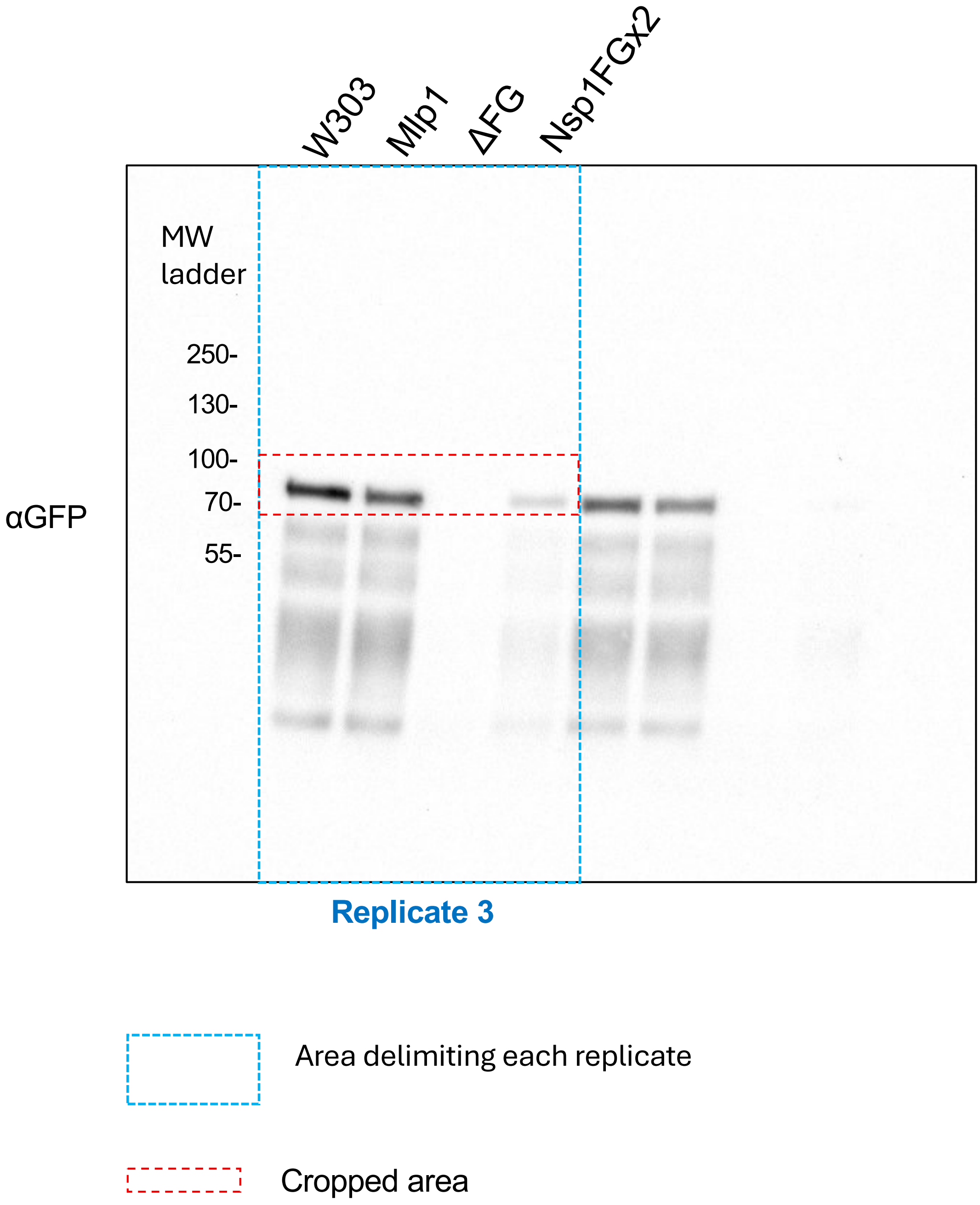
W303
Mlp1
ΔFG
Nsp1FGx2
MW
ladder
250-
130-
100-
70-
55-
αGFP
Replicate 3
Area delimiting each replicate
Cropped area

ED Fig. 6b

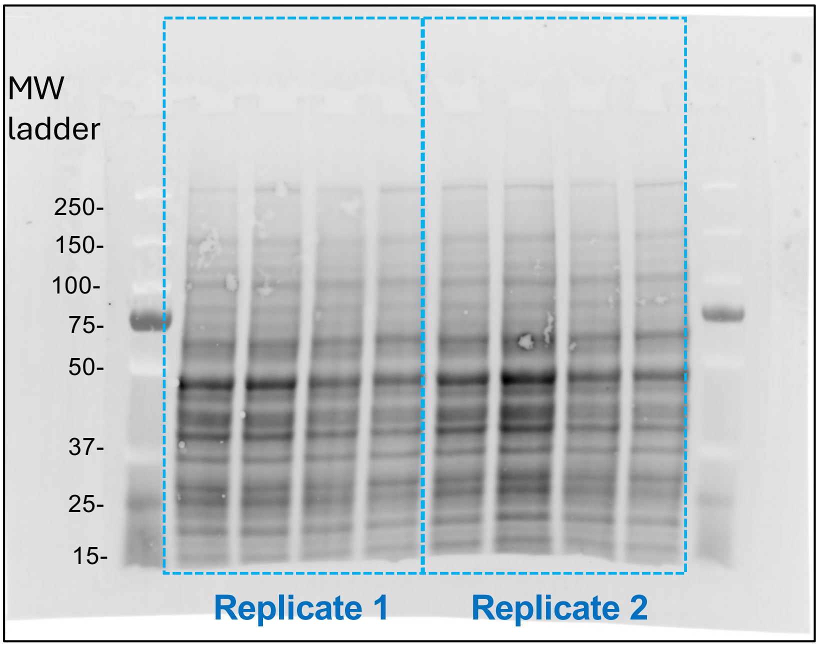

Stain free membranes

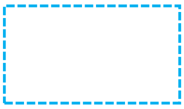

Area delimiting each replicate

ED Fig. 6b

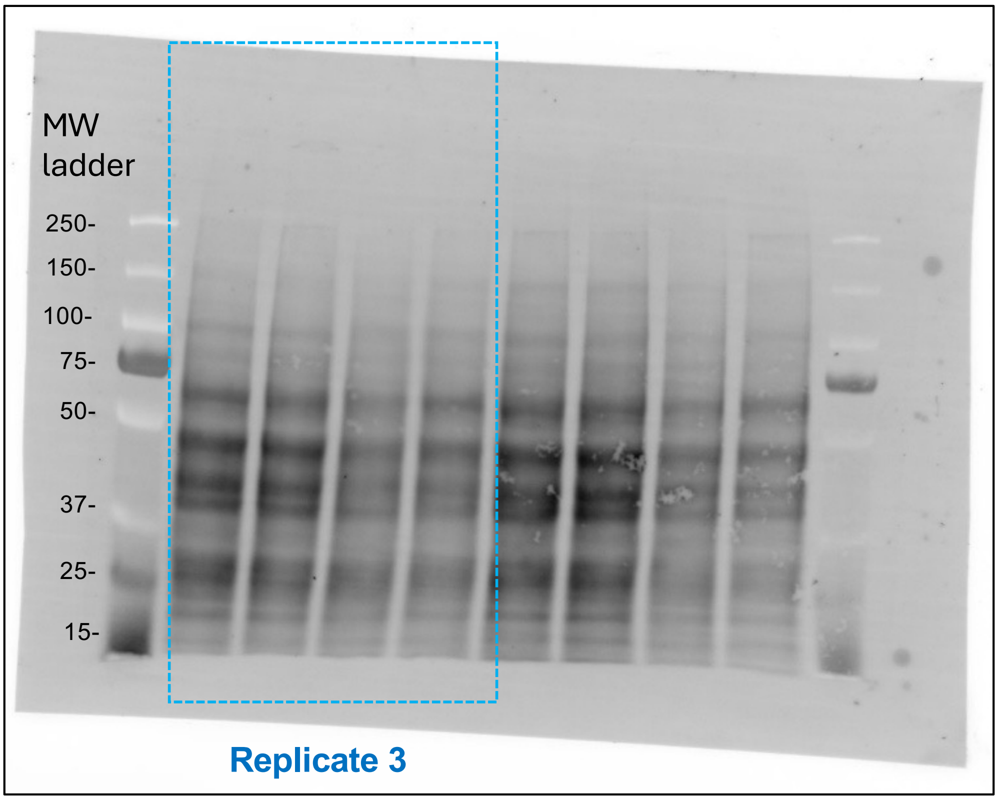

Stain free membranes

Area delimiting each replicate
